# Correction: NADPH oxidase mediates microtubule alterations and diaphragm dysfunction in dystrophic mice

**DOI:** 10.7554/eLife.84169

**Published:** 2022-10-17

**Authors:** James Anthony Loehr, Shang Wang, Tanya R Cully, Rituraj Pal, Irina V Larina, Kirill V Larin, George G Rodney

**Keywords:** Mouse

Loehr JA, Wang S, Cully TR, Pal R, Larina IV, Larin KV, Rodney GG. 2018. NADPH oxidase mediates microtubule alterations and diaphragm dysfunction in dystrophic mice . *eLife* **7**:e31732. doi: 10.7554/eLife.31732.

Published 30 January 2018

As we were analysing data for an additional manuscript we found that we made a mistake in presenting the unit (or the scale) in Figure 3C. The unit (or scale) for Figure 3C should be 10^4^ Pa instead of Pa. This is because the stress values provided in the stretch data have a unit of N/cm^2^ (Figure 3B) instead of N/m^2^ (or Pa), and 1 N/cm^2^=10^4^ N/m^2^ (or Pa). We have corrected this error. This correction does not change the results or affect the scientific conclusions of the original report.

The corrected Figure 3C is shown here:

**Figure 1:**
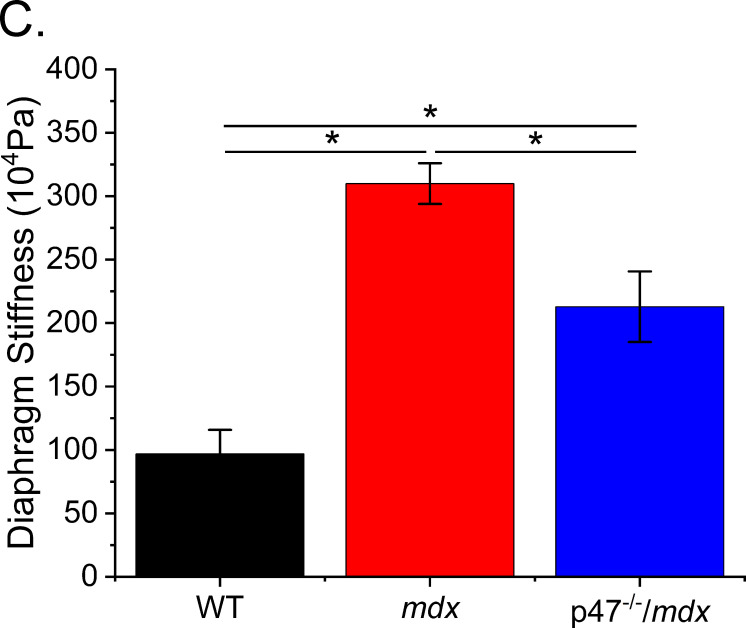


The originally published Figure 3C is shown for reference:

**Figure 2:**
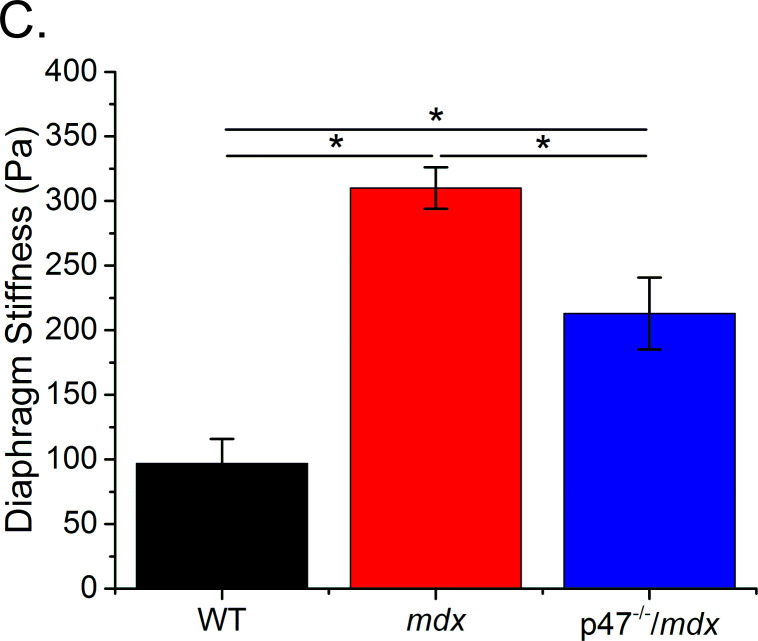


The article has been corrected accordingly.

